# Phages in the infant gut: a framework for virome development during early life

**DOI:** 10.1038/s41396-021-01090-x

**Published:** 2021-08-20

**Authors:** Michael Shamash, Corinne F. Maurice

**Affiliations:** Department of Microbiology & Immunology, McGill University, Montreal, QC Canada

**Keywords:** Bacteriophages, Bacteriology, Microbiome

## Introduction

The human gut is home to trillions of microorganisms including prokaryotes, eukaryotes, and viruses which each have integral roles in maintaining the stability and resilience of this ecosystem, as well as the health of their human host. Bacteriophages (phages) make up the majority of the viral fraction in the gut and are constantly predating on their bacterial hosts [1]. During the first few years of life, the infant gut microbiota follows a defined successional pattern where both bacterial and phage communities experience consistent shifts in overall abundance and taxonomic composition [2–5]. The gradual maturation of gut bacterial communities during early childhood is well described and central to an individual’s healthy development. Perturbations of the gut microbiome early in life, such as antibiotic usage, have been associated with a myriad of health outcomes [6–8]. In contrast, phage dynamics in the infant gut are not as well characterized, and thus, their potential roles in gut homeostasis and physical and cognitive development remain unclear [3, 9, 10]. Mostly known as agents of bacterial lysis, phages represent an important reservoir of genetic diversity which can confer selective metabolic, immune, and evolutionary advantages to bacterial hosts [11]. How phages replicate also has distinct consequences on bacterial communities, and by extension the human host [11–14]. Assessing the role and contribution of phages to the gut microbial ecosystem is thus necessary to gain insight into the factors shaping microbial succession, better understand the impacts of perturbations, and ultimately to intervene. In this perspective, we first summarize current knowledge on the development of the infant gut microbiome, focusing on phage-bacteria interactions, and speculate on understudied aspects. We then propose a theoretical framework governing the development of the gut virome in early childhood, which is dependent on both phage and bacterial densities and diversity. We conclude by suggesting the use of newly developed single-cell and bioinformatic approaches to decipher the complex network of microbial interactions occurring in the infant gut.

### Development of the gut bacteriome and virome in early childhood

**Fig. 1:**
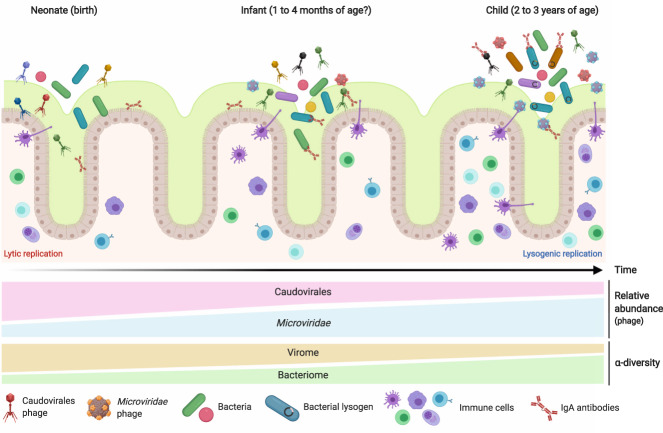
Development of the infant gut microbiome from birth to early childhood. Initial phage colonizers can be detected during the first week of life, forming a community which is of high diversity and dominated by Caudovirales phages. Phages rapidly increase in abundance during the first few months of life, a process which is also associated with a global reduction in virome ɑ-diversity. During the same time, there is a steady increase in bacterial abundance and ɑ-diversity. The factors driving this shift in phage community composition remain unknown. Possible contributing factors include loss/gain of bacterial taxa over this period, development of the infant’s immune system (depicted with increasing number of immune cells in the tissue adjacent to the intestinal lumen), changes in bacterial and viral densities, and acquisition of new phages from environmental sources. Both the phage and bacterial fractions of the gut microbiome reach an adult-like composition by 2–3 years of age.

During the progression from early infancy to toddler years (2–3 years old), there is a marked shift in both gut bacterial and viral community composition and abundances (Fig. 1). The gut bacteriome decreases in diversity during the first week of life (attributed to environmental bacteria unable to colonize the newborn’s gut) [15] before increasing steadily in diversity and abundance [3, 4, 16] (Fig. 1). This community is first dominated by facultative anaerobes of the Firmicutes (*Streptococcaceae* and *Staphylococcaceae*) and Proteobacteria (*Enterobacteriaceae*) phyla. In healthy breastfed individuals, members of the Actinobacteria phylum (primarily *Bifidobacteriaceae*) are maintained at high abundances during the first year of life. With a solid diet, a diverse community of obligate anaerobes, including Bacteroidetes (*Bacteroidaceae*) and additional Firmicutes (*Lachnospiraceae* and *Ruminococcaceae*), begins to form and stabilizes by 2–3 years of age. At this point, the Bacteroidetes- and Firmicutes-dominated bacterial community resembles the adult gut microbiome [2, 17]. Formula-fed infants show an accelerated maturation of their gut bacterial communities upon the cessation of breastmilk [18].

With regards to the gut virome, the current consensus is that the neonatal gut is free of viruses at birth and quickly becomes colonized by phages within the first week of life. These initial phage colonizers seem primarily derived from induced prophages of pioneering gut bacteria [19], themselves obtained from environmental sources such as the birth canal [6], maternal gut microbiome [20], or from breastfeeding [21, 22]. For instance, several prophage-harboring *Bifidobacterium* sp. which are vertically transmitted from maternal breast milk to the infant have been shown to be induced in the gut [22].

Contrary to the gut bacteriome’s overall trend of increased diversity during early life, the diverse and rich phage-dominated virome slowly decreases in richness with age until reaching an adult-like abundance and taxonomic profile by 2–3 years of age [3, 23] (Fig. 1). The infant gut virome is predominantly first composed of members of the order Caudovirales, and by 2 years of age the *Microviridae* family of virulent phages are most prevalent, similar to adult gut viromes [24] (Fig. 1). This inverse relationship between Caudovirales and *Microviridae* relative abundances in the infant gut has been observed in multiple independent studies [3, 19, 20, 25]; however, the ecological factors driving this shift remain unknown. For their part, the ubiquitous family of crAss-like phages, including the prototypical crAssphage (p-crAssphage), a group of lytic phages infecting members of the phylum Bacteroidetes, first appear at 1–3 months of age and become widespread among children 2–5 years old [19, 23, 26]. This delayed appearance is likely related to the later acquisition of the host bacterial taxa (specifically *Prevotellaceae* and *Bacteroidaceae*) during gut microbiome development [2, 27]. Two groups have investigated the colonization and vertical transmission of p-crAssphage and crAss-like phage with conflicting results. Several complete genomes with high homology to p-crAssphage were detected in two cohorts of 20 Irish and 40 Malawian infants irrespective of birth mode [28]; whereas a more extensive study of 143 mother-infants pairs showed that only infants which were born vaginally harbored crAss-like phages, suggesting vertical transmission [26]. Recently, a temperate crAss-like phage in adult gut metagenomes was detected [29], raising the possibility that the first crAss-like phages detected may also be derived from induced crAss-like prophages. Thus, additional work is warranted to determine the dynamics of crAssphage colonization in the infant gut.

Most virome studies to date have relied on database-dependent approaches to process viral sequencing data, leaving up to 65% of viral sequences taxonomically unclassified and excluded from further analysis (up to 90% of sequences in adults) [9, 30]. Furthermore, characterized phage genomes deposited to publicly available databases are biased in terms of their diversity and described hosts. The majority of the ~4100 Caudovirales phages deposited in NCBI RefSeq (accessed March 26, 2021) have either Gammaproteobacteria, Actinobacteria, or *Lactobacillales* as their host, all of which are highly abundant in the infant gut. In contrast, there are only about 70 *Microviridae* phages deposited to this reference database, highlighting that this phage family and its host range are significantly understudied. In addition, few Caudovirales phages infecting members of the Bacteroidetes phylum have been described, possibly leading to under-detection of these phages once Bacteroidetes become abundant in the gut. It is thus probable that the observed inverse relationship between Caudovirales and *Microviridae* with age is an artifact associated with current public databases.

Recent advances in viral metagenomics have led to the development of database-independent approaches, such as the use of all-versus-all comparisons to form viral clusters by shared gene content [31]. Applying this technique to over 1300 previously unclassified viruses deposited in NCBI RefSeq yielded confident genus-level host assignments for 60% of the viral sequences [31]. Depending on the cutoffs, viral clusters can be equivalent to genus- or family-level taxonomy ranks [32]. This allows for a more comprehensive study of all detected phages by studying them at the viral cluster level, instead of excluding sequences which do not align to reference databases altogether [24, 31, 33]. Using a clustering approach combined with strict criteria for decontamination of virome sequences (see [24]) would allow us to gain a more complete understanding of viral succession during early life. Additionally, studies should investigate functional genes in phage genomes, as they are key in determining host cell phenotypes during infection [11] and can provide additional insight into the active roles of phages in the gut. Finally, future studies should evaluate how widespread the mechanism of phage colonization-by-prophage-induction truly is.

### Phage-bacteria dynamics in the infant gut: a moving picture

As described above, the rapid turnover of bacterial taxa during the first years of life is likely a significant contributing factor in the development of the gut phage community – as new hosts appear and existing hosts disappear, so would their associated phages [34]. Changes in the phage community could also drive changes in the gut bacterial communities. For example, the direct interactions between phages and the immune system may select for, or against, certain phages through antibody sequestration [8, 35, 36], which may then result in downstream effects on gut bacterial communities. Furthermore, phages from environmental sources could also remodel gut microbial communities and become stable members of the community if their bacterial hosts are present [13].

The first description of the infant gut virome using DNA microarrays revealed a rapid turnover of phages during the first 2 weeks of life [9]. These findings provided preliminary evidence for kill-the-winner (KTW) dynamics between bacteria and their viruses. KTW dynamics are a type of Lotka-Volterra dynamics, where lytic phage infection of the most abundant bacteria leads to rapid, often cyclical, shifts in bacterial species abundance and diversity [37]. Since then, two additional metagenomic studies of the neonatal gut have yielded similar findings, tracking the interactions between bifidoprophages and their *Bifidobacterium* hosts [38], and several *Staphylococcus epidermidis* strains and their phages in the gut of a preterm infant [10]. These two studies used correlational analyses between phage and bacterial relative abundances to infer phage-host pairings, a method which is accurate only 10% of the time [39], implying that there may be mis-associations and underestimations of pairings. Furthermore, the use of relative abundances in determining dynamics from metagenomic data should be used cautiously, as a host (or phage) can vary dramatically in relative abundance without any change to its absolute abundance, and vice versa [40]. This is especially relevant in the infant gut, where absolute abundances of phage and bacteria are both in constant flux throughout development.

By looking for cyclical patterns in the relative abundances of phage and bacteria, one may be biased to discovering KTW-like interactions, and only among the most abundant phage-bacteria pairings. Indeed, to detect interactions associated with antagonistic coevolution, one would need to study the genomes of both the bacteria and their phages over time [41]. These highly dynamic interactions include both arms race dynamics (ARD) and fluctuating selection dynamics (FSD). Arms-race dynamics are defined as the directional evolution of bacteria and phage toward phenotypes associated with increasing resistance or infectivity, respectively [42–45]. This contrasts with fluctuating selection dynamics, where negative frequency-dependent selection leads to fluctuations in phage and bacterial genotypes and levels of resistance [42, 46, 47]. These models of phage-bacteria interactions (KTW, ARD, and FSD) do not exist in a vacuum, and in complex communities such as the mammalian gut, they likely co-occur or replace each other over time [1]. Longitudinal genomic data to determine which types of interactions take place in the infant gut could include mutations in phage tail fiber or accessory genes to expand host range, or acquisition of new bacterial CRISPR spacers, amongst others [41, 48–50]. Additionally, longitudinal analysis of the bacterial metatranscriptome could be used to study changes in surface protein expression (and thus, phage receptors), as well as exploring the diverse mechanisms bacteria have evolved in response to phage infection (see [51] for an overview). Determining interaction networks in complex communities such as the gut microbiota from correlation data alone is a daunting task which should always be followed with additional in vitro or in vivo confirmatory experiments with simplified communities or isolates, where possible [52].

### A framework for gut virome development during early life

Many non-mutually exclusive pressures could be driving the switch from a primarily lytic gut virome in childhood to a more stable one dominated by integrated prophages in adulthood. For instance, prophage-encoded genes can provide metabolic benefits to their host bacteria, such as carbon and xenobiotics metabolism pathways or antibiotic resistance genes [12, 53, 54]. Prophage-encoded virulence factors can promote pathogen survival, as with *Vibrio cholerae* and its prophage CTXΦ [55]. Prophages can also increase bacterial competitiveness in complex communities, as described with the prophages of *Lactobacillus reuteri* in the gut [56]. Furthermore, lysogeny can suppress lytic phage infection, and thus increase community stability, through superinfection immunity, whereby prophages protect their hosts from secondary infection through prophage-encoded modifications to cell-surface proteins and receptors [57–59]. Finally, coinfections by temperate phages, a phenomenon that becomes more prevalent with increasing phage densities, have been shown to promote lysogenization in model phage-bacteria systems, such as lambda phage and *Escherichia coli* [60, 61]. Coinfections increase the expression of transcriptional repressors of the lytic pathway, activating the cascade responsible for genome integration [60]. Emphasizing the importance of phage coinfections in complex microbial communities, a recent biophysical model quantified the probability of lysogen formation in marine and human gut microbiomes [61]. This model considers bacterial host densities, lysogenic commitment times, phage-bacteria encounter rates, phage infection efficiencies, and temperate phage concentrations, which all ultimately determine the number of coinfections at any given moment. The authors demonstrated that the high frequency of lysogeny in the adult human gut can be explained by high phage and bacterial densities, as well as relatively high phage adsorption rates, resulting in the generation of approximately two trillion lysogens daily. This model supports piggyback-the-winner (PtW) phage-bacteria dynamics, proposed to be widespread in soil and host-associated environments [62, 63], whereby lysogeny dominates at high bacterial abundances and growth rates as temperate phages passively exploit their bacterial hosts.

**Fig. 2:**
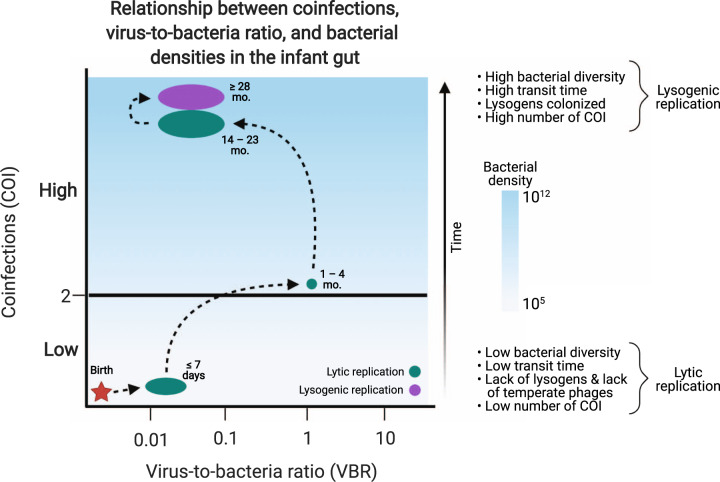
Proposed framework governing the development of the gut virome during early life. At birth, the neonatal gut virome is quickly colonized by induced prophages of pioneering bacteria (red star). Low bacterial abundances do not allow for the lysogenic maintenance of these phages (low number of coinfections, COI) and environment-derived lytic phages as well as induced prophages dominate the virome during the first few months of life. As bacterial and viral densities increase in the infant gut, so does the virus-to-bacteria ratio (VBR), increasing the number of COI per bacterial cell. However, these COI are primarily from lytic phages, due to the low number of bacterial lysogens during early life, and thus there is no pressure for lysogeny resulting from these coinfections. As the infant matures and changes diets, a diverse community of prophage-harboring obligate anaerobes begin to colonize the gut. The gut virome now contains temperate phages from a more diverse and abundant community of bacterial lysogens which coinfect their bacterial hosts and in doing so, maintain overall lysogenic stability in the system, as seen in adult gut microbiomes. Additional pressures can also maintain lysogeny (see main text for details).

To apply the concepts of phage coinfection to the developing gut, bacterial and phage densities during this period must first be considered. Virus-like-particles (VLPs) are often undetectable in feces during the first days of life (median 17 h after birth), while the number of copies of the bacterial 16S rRNA gene per gram feces, when detectable, is ~3 × 10^6 ^g^−1^, resulting in a very low virus-to-bacteria ratio (VBR) [19]. VLPs become detectable in infant feces by microscopy as of 1 week of age [9]. Between 1 and 4 months after birth, both VLP counts and bacterial 16S copy number each stabilize at 10^9 ^g^−1^, leading to a VBR of ~1 [19]. By 2 years of age and onwards, the average abundances of VLPs and bacteria are 10^10 ^g^−1^ and 10^12 ^g^−1^, respectively, resulting in a decreased VBR of about 0.01 [7]. These low VBRs, also found in adulthood, are typically suggestive of lysogeny[64]. Extrapolating these VBR and bacterial density estimates results in increasing numbers of coinfections per bacterial cell as infants age (Fig. 2).

The prevalence of temperate phages and bacterial lysogens in the developing infant gut is currently unknown, yet is a key factor to consider in the coinfection model. In the adult gut, the majority of active temperate phages are predicted to have *Lachnospiraceae* (Firmicutes phylum) and *Bacteroidaceae* (Bacteroidetes phylum) hosts [12, 65–67], which only stabilize in the infant gut after 2 years of age. In the murine gut, it is estimated that 64% of the lysogens are among the Firmicutes (Clostridia class) and 20% among the Bacteroidetes (Bacteroidia class) phyla, while Actinobacteria, which are very abundant in the infant gut, are more prevalent in the non-lysogenic group (9% of all non-lysogens) [68]. One can speculate that the lack of Clostridia and Bacteroidia in the neonate gut would limit the existence of a lysogen-dominated community, despite the increased probability of coinfections.

Considering increasing coinfections with age and the initial lack of bacterial lysogens in the infant gut, we propose a theoretical framework wherein temperate phages are initially fundamental in seeding the gut virome through prophage induction of pioneering gut bacteria, while lytic phages (acquired during birth and later from environmental sources) are key actors in modulating the gut bacteriome throughout the first few years of life.

During the first months of life, coinfections by induced temperate phages of the seeding gut bacterial taxa could lead to an increased pressure for lysogeny; however, the low absolute abundances of both bacteria and phages during this period is unlikely to sustain a sufficient number of coinfections (Fig. 2). Coinfections of bacterial hosts by lytic phages during this same early period would have no direct effect on community-wide lysogeny levels, as these phages are strictly lytic. Once the acquisition of sufficient lysogenic hosts and their temperate phages (as prophages) has occurred, likely after the second year of life, coinfections by these temperate phages becomes a driving force for lysogeny in the community. Resilience of a stable lysogen-dominated community would then be maintained through a wide variety of prophage-encoded genes and selective pressures, as described previously.

This could explain in part why successful remodeling of infant gut-derived bacterial communities by gut phages is age-dependent, with successful in vitro remodeling outcomes using samples from children less than 2 years old [7]. With this framework in mind, we would expect the relative abundance of free induced temperate phages to be high at birth (as described in [19]), likely due to their active lytic replication, and decrease with age as the number of coinfections by temperate phages increases. Eventually, the abundance of free temperate phages will plateau, and prophage induction (spontaneous or due to stressors) would be responsible for seeding the lysogenic portion of the virome. This age-dependent decrease in free temperate phage abundance was observed in a cohort of healthy and stunted children [7], supporting our theoretical framework. A very recent study also suggested that over 94% of prophages identified in metagenome samples are active in infants one year of age [69].

Two abiotic factors could also shape phage-bacteria dynamics during early life: intestinal transit times and the gut mucosal barrier. Intestinal transit time steadily increases during the first 4 years of life and is strongly associated with gut microbiome composition, diversity, and metabolism [70, 71]. Its effects on gut virome diversity and composition have yet to be explored; however, we hypothesize that increasing colonic transit times allow for more interactions between phages with lower adsorption rates and their hosts. The gut mucosal barrier for its part allows for spatial distribution of bacteria in the lumen/on the mucus surface and phages deeper in the mucus because of their adherence to mucin glycoproteins [72, 73]. This spatial distribution is suggested to be important in determining lytic and lysogenic interactions [74], with lysogeny being more common at the outer mucosal surface [62], possibly as a result of increased coinfections.

Driven by currently unavailable data, the framework we present is difficult to test directly. The VBRs we calculated reflect an overall ratio in the gut and do not consider host specificity, and the potential roles of phage diversity in gut virome maturation and community dynamics also remain to be considered. Finally, other phage replication cycles besides strictly lytic or lysogenic exist [75]. However, simplifying phage-host interactions to lytic or lysogenic in the developing gut provides a strong framework to then consider other phage replication strategies.

Validating our proposed framework in the developing gut using longitudinal cohorts, combined with both bioinformatic and in vitro approaches, will provide a better understanding of how and when lysogeny begins to dominate. The combined use of absolute phage and bacterial abundances with metagenomic and experimental data to infer host pairings will allow for phylum or family-level VBRs, and thus more accurate coinfection values. Additionally, metagenomic data should be used to determine the prevalence of lysogenic bacteria and their dynamics during early life. In vitro systems seeded with infant gut samples could be used to evaluate the impact of mucus and transit times on phage-bacteria interactions in a controlled setting [76].

If lytic interactions do indeed dominate the newborn gut, this could represent an optimal time frame to use phages to remodel gut bacterial development, known to be impaired in childhood malnutrition, stunting, and in necrotizing enterocolitis [7, 13, 77]. Such an approach could restore the expected healthy successional trajectory of the gut microbiome. Given the potential of phage therapy to target specific bacterial taxa or reduce symptoms of a disease or perturbation through entire virome transplants in adults and mice [13, 78, 79], phage remodeling of gut bacterial communities for gut microbiome development is an appealing approach. Yet, additional work is needed to fully understand the impacts of using fecal virome transplants and selective phage cocktails, especially if bacteria-phage interactions in the infant gut are distinct from the adult one. It is especially interesting to consider the potential role of lysogeny in limiting the effectiveness of lytic phages in restructuring the adult gut microbiome. In vitro experiments with distinct phage communities or a cocktail of phages of interest combined with mouse models of early life development are imperative to assess the feasibility and outcomes of this phage-led remodeling.

### From correlations to associations: determining phage-bacteria interactions in the infant gut

**Fig. 3:**
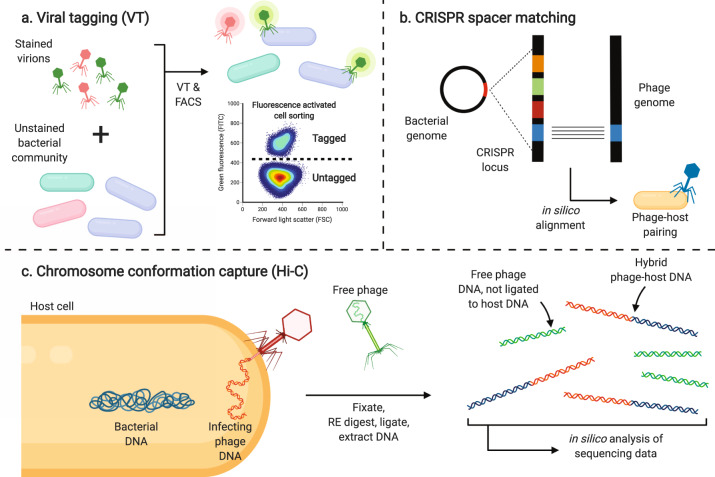
Modern techniques for determining bacteriophage-bacteria interactions. **a** Viral tagging (VT) allows for the study of phage-bacteria interactions at the single-cell level. Both phage and bacterial communities are derived from the same original sample. Virions are stained with a fluorescent nucleic acid dye and are incubated with an unstained bacterial community prior to fluorescence-activated cell sorting (FACS). The virally tagged bacterial population has increased fluorescence over its non-tagged counterpart and individual phage-bacteria pairs from this population are sorted and further sequenced. **b** CRISPR spacer matching is a strictly computational approach to determining phage-bacteria pairs. CRISPR loci in bacterial metagenome-assembled genomes are identified and aligned to phage contigs from the same sample. Alignment of these short spacer sequences to a phage genome is indicative of previous infection of the bacteria by the phage and thus a phage-host pairing is established. **c** High throughput chromosome confirmation capture (Hi-C) takes advantage of the close proximity of phage and host bacterial DNA during infection to identify phage-host pairings de novo. Samples are first cross-linked, digested by restriction endonuclease (RE) before being re-ligated. DNA fragments near one another will form a hybrid upon re-ligation, as is the case for infecting phage DNA with host DNA. DNA from free intact phages will not be ligated to any bacterial DNA. Re-ligated DNA is then sequenced to determine phage-host pairings.

In order to understand how phage and bacteria coevolve in the developing gut, it is necessary to assign phage-host pairings with a degree of certainty greater than that obtained from correlational analyses alone. Despite the rapid increasing number of assembled viral sequences, it remains difficult to associate phages with their hosts at high levels of taxonomic resolution, preventing any meaningful microbial population dynamics interpretation. Excitingly, recent advances in sequencing technologies, single-cell techniques, and analytical tools are allowing the field to move beyond strict correlational analyses and identify novel phage-bacteria pairings. For instance, viral tagging (VT) takes advantage of the physical interaction between a virus and its host during the initial steps of infection [65, 80] (Fig. 3a). Fluorescently stained viral populations are incubated with an unstained bacterial population. Bacterial cells tagged with a fluorescent phage are then sorted individually by fluorescence-activated cell sorting. The pair’s genomes (bacterium and attached phage) can then be sequenced and characterized to determine phage-host pairings. For many virus-host pairings described to date, VT adsorption is equivalent to infection [80], although this remains to be verified in the human gut. Non-specific binding between phage and bacteria must also be assessed, as this can lead to false-positive results [65].

Host-phage pairings can also be established by CRISPR spacer matching in silico (Fig. 3b). Bacterial CRISPR systems are a type of adaptive immunity, allowing for acquisition of resistance to an infecting phage. Briefly, invading viral DNA is recognized by endogenous bacterial enzymes, and a short 25–75 bp fragment of the foreign DNA is excised and inserted into the CRISPR locus in the bacterial genome, forming a new CRISPR spacer. Researchers can take advantage of this memory of previous infections by using computational methods which align CRISPR spacers from bacterial genomes to phage sequences obtained from the same sample [39]. With stringent cutoffs for nucleotide identity and alignment length, sequences which align can be classified as phage-host pairings. Although CRISPR spacer matching is very specific, it is not very sensitive and thus can drastically underestimate active phage-bacteria interactions. This lack of sensitivity can be attributed to the fact that only 40% of bacteria encode a CRISPR system [39]. Additionally, many CRISPR spacers do not match known sequences [39] and may result from infection by a phage that has been lost from the ecosystem. Furthermore, the short length of spacer sequences could lead to alignment to a biologically-incorrect target sequence, generating a false-positive result. Addressing these challenges by evaluating their individual impact in various controlled experiments and ecosystems is key to validating these techniques. Oligonucleotide usage profiles (k-mer frequencies) and codon usage patterns have also been used to deduce phage-host pairings in silico, with varying levels of success [39].

High-throughput chromosome conformation capture (Hi-C) is another approach which may be used to determine pairings de novo. This technique involves the cleavage of cross-linked DNA and its subsequent re-ligation to determine which fragments of DNA were in close spatial proximity, which can be interpreted as a pairing of phage and host DNA in the same cellular compartment (Fig. 3c). Hi-C assays have unveiled complex interaction networks in human gut microbiome studies [81]. Two key limitations of the method are the varying efficiencies of the assay itself due to differing cell wall components, making it easier or more difficult for restriction enzymes to enter the cell, as well as the possibility of strain interference which may fragment assemblies [82]. Additionally, this method is unlikely to detect RNA phages altogether as the enzymes used throughout the assay require DNA molecules as substrates.

Finally, culturomics have allowed for the high-throughput isolation of previously unculturable bacterial taxa from environmental samples, including the human gut [83]. This approach could significantly increase our capacity to manipulate and isolate gut phages, while screening for optimal growth conditions in a high-throughput manner. A crossover between viral tagging and culturomics has been proposed to identify, isolate, and purify unknown phages from environmental samples [84]. While culturing is the “gold standard” for phage isolation and characterization, this method can prove to be quite time and resource intensive when scaling up to a complex community.

Applying these methods to samples obtained throughout early life will allow us to better understand the dynamic relationship between phages and their bacterial hosts at scales which were previously not feasible. To date, many studies have focused primarily on either gut phage or bacterial communities at a single time point, and their individual associations with health or disease. By moving beyond single whole-community snapshots to longitudinal sampling of both phage and bacterial communities, we can determine the active members of these populations, how these communities coevolve together over time and under different conditions (change in diet, age, disease status, etc.), bringing us one step closer to understanding how these microorganisms shape infant growth and health.

## Concluding remarks

Research on the gut microbiome in early childhood has focused primarily on either the bacterial or viral populations, but not how they interact during this key developmental period. Factors driving shifts in phage community structure in child development remain unknown, despite the wealth of data demonstrating that phages are crucial regulators of bacterial communities. We propose a theoretical framework for infant gut virome development where upon birth, there is a lack of bacterial lysogens and too low phage and bacterial densities to sustain lysogeny. The lack of bacterial lysogens during this period also allows for a lytic phage-dominated virome to persist alongside induced prophages from pioneer bacteria. By 2 years of age, both phage and bacterial numbers increase and stabilize, including the number of bacterial lysogens in the community. This increased microbial density and abundance of temperate phages creates an environment where a significant number of coinfections per bacterial cell, a driving pressure for lysogeny, can occur, resulting in community resilience to phage-driven alterations. Combining established molecular and bioinformatic techniques, namely viral tagging, CRISPR spacer matching, and Hi-C assays, in addition to culturomics, will allow deeper understanding of these individual phage-bacteria interactions. Ultimately, by understanding the dynamics of the human gut virome during infancy, specific therapeutic windows for phage-driven remodeling of the gut microbiome can be identified and applied therapeutically to restore the successional dynamics of the bacterial community.
